# Autopsy Proven Peripheral Nervous System Neurolymphomatosis Despite Negative Bilateral Sural Nerve Biopsy

**DOI:** 10.3389/fneur.2013.00197

**Published:** 2013-12-06

**Authors:** Adolfo Ramirez-Zamora, Sarkis Morales-Vidal, Jasvinder Chawla, José Biller

**Affiliations:** ^1^Department of Neurology, Albany Medical Center, Albany, NY, USA; ^2^Department of Neurology, Maguire Center, Stritch School of Medicine, Loyola University Chicago, Maywood, IL, USA; ^3^Edward Hines, Jr. VA Hospital, Hines, IL, USA

**Keywords:** neurolymphomatosis, non-Hodgkin lymphoma, peripheral neuropathy

## Abstract

Neurolymphomatosis (NL) refers to a lymphomatous infiltration of peripheral nerves associated with central nervous system or systemic lymphoma, or alternatively, neurodiagnostic evidence of nerve enhancement and/or enlargement beyond the dural sleeve in the setting of primary central nervous system lymphoma or systemic lymphoma. NL is a rare complication of systemic cancer with heterogeneous clinical presentations and an elusive diagnosis. Diagnosis usually requires the demonstration of infiltrating malignant lymphocytes in the peripheral nerve. Infiltration of brain parenchyma, meninges or Virchow–Robin spaces is characteristic of systemic disease at autopsy. We describe a patient presenting with biopsy negative NL affecting exclusively the peripheral nervous system at autopsy.

## Introduction

Neurological complications of cancer are protean and can involve any area of the neuroaxis through direct local invasion or distant paraneoplastic effects. One of the least well-understood and recognized complications of cancer, particularly with non-Hodgkin’s B-cell lymphomas (NHL) or primary central nervous system lymphoma (PCSNL) is neurolymphomatosis (NL) (1). NL refers to a lymphomatous infiltration of peripheral nerves associated with central nervous system (CNS) or systemic lymphoma. Alternatively, the presence of nerve enhancement and/or enlargement beyond the dural sleeves in neurodiagnostic studies or intraoperative evidence nerve invasion in the setting of PCNSL systemic NHL is diagnostic of the disease (2). NL is mainly caused by diffuse large B-cell lymphomas, usually in the setting of widespread NHL, but it may be the first or the sole manifestation of relapse. NL patients commonly present with lymphomatous infiltration of peripheral nerves, meninges, Virchow–Robin spaces, and brain parenchyma but with clinical features of isolated or multifocal nerve dysfunction (2–4). The recognition of this uncommon disease is challenging, but critical to provide adequate management. Exclusive infiltration of peripheral nerves by lymphoma cells is rare with only two patients with NL confined to the peripheral nervous system (PNS) reported in the literature (5, 6). The authors suggested a possible lymphoma-specific interaction between immunologic ligands and endothelial receptors at the blood-nerve barrier as the potential cause.

We report the clinical, radiographic, and pathological findings of an additional patient diagnosed with NL confined to the PNS.

## Case Presentation

A 62-year-old right-handed man with a past medical history remarkable for coronary artery disease was transferred to our institution after worsening weakness and sensory changes involving his left hand. Family and social history were unremarkable. He initially presented to an outside hospital after developing flu-like symptoms for a week, followed by worsening weakness, numbness, and tingling of the second and fifth digits of the left hand. Neurological symptoms rapidly progressed to affect his entire left upper extremity. Electromyography and nerve conduction studies (EMG/NCVs) showed demyelinating features in multiple left upper extremity nerves. Neurological examination showed normal cognition and cranial nerves function. There was marked left arm flaccid weakness and mild distal right hand muscle weakness. Sensory examination showed decreased light touch and pin-prick on the left upper extremity and evidence of mild distal lower extremities weakness and areflexia. General examination demonstrated marked splenomegaly and generalized lymphadenopathy.

He was diagnosed with mononeuritis multiplex, and trials of immune-modulating agents [prednisone, intravenous immunoglobulin G (IVIG), and plasmapheresis (PLEX)] resulted in partial and only transient improvement of sensorimotor symptoms. A bone marrow biopsy identified a large B-cell NHL.

**Figure 1:**
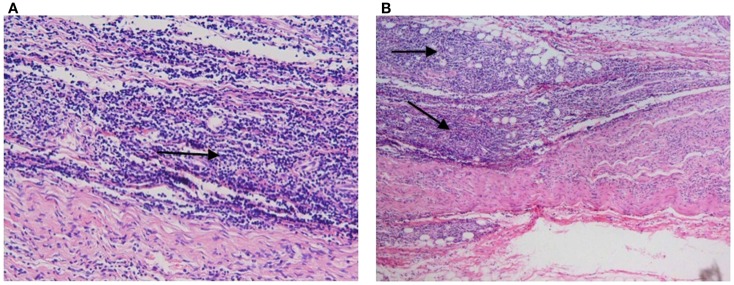
**Hematoxylinand eosin (H&E) stains**. Post-mortem examination revealed diffuse and monotonous propagation of lymphoma cells (arrows) infiltrating peripheral nerves without involvement of the brain parenchyma or meninges.

Magnetic resonance imaging (MRI) of the cervical spine and brachial plexus showed no evidence of tumoral infiltration of the cervical cord or brachial plexus. Cerebrospinal fluid (CSF) examination was performed in two different occasions due to concerns of meningeal infiltration yielding with normal results. The patient was subsequently started on induction chemotherapy including two cycles of cyclophosphamide, doxorubicin, vincristine, prednisone, and rituximab, with partial improvement in strength and sensory symptoms. Two months later he developed worsening weakness in the contralateral hand and mild weakness on the distal left lower extremity. Muscle stretch tendon reflexes were absent. Repeat EMG/NCS showed findings associated with demyelinating polyneuropathy and lumbar radiculopathy with denervation potentials. A sural nerve and muscle biopsy showed a mixed axonal and demyelinating neuropathic process with no specific inflammatory changes in muscle specimen. Over the following month he developed gait difficulties and frequent falls as a consequence of worsening weakness in lower limbs. He also reported hyperesthesia and marked pain on his right arm, associated with progressive muscle weakness. No cranial nerve involvement was observed. He received five PLEX and high-dose steroids with limited improvement. Repeated infusion of IVIG did not improve his motor/sensory deficits. He subsequently completed another cycle of chemotherapy with good laboratory and imaging response regarding his lymphadenopathy and splenomegaly. Follow-up CSF examination was normal. Flow cytometry was indeterminate. Contralateral sural nerve and quadriceps muscle biopsy showed non-specific pathological findings. Repeated CSF analysis showed mild elevation of protein content. He received a cycle of intrathecal methotrexate due to concerns of meningospinal tumor infiltration. The patient was restarted on chemotherapy and completed six courses of CHOP-R (Cyclophosphamide, Hydroxydaunorubicin, Oncovin, Prednisone) with only minor improvement on his neurological deficits. Approximately 7 months following onset of symptoms, he developed binocular horizontal diplopia due to a left third nerve palsy followed by left periorbital discomfort and numbness on the left chin, consistent with “numb” chin syndrome. He continued to worsen and finally develop a flaccid, areflexic quadriplegia, with muscle atrophy without fasciculations. Extensive immunologic, metabolic, and infectious ancillary investigations were normal. Repeated brain [including Fast Imaging Employing Steady-state Acquisition (FIESTA) protocol], spine, and brachial plexus imaging showed no abnormalities. Unfortunately, the patient developed multiple systemic complications requiring intubation, tracheostomy, and prolonged intensive care. He eventually died due to sepsis, neutropenia, and multiple organ failure. At autopsy, lymphoma cells infiltration was demonstrated in multiple cranial nerves including left third, sixth, trigeminal nerve root, and bilateral facial nerves. Widespread, patchy, lymphoma infiltration in peripheral nerves, and distal portions of the spinal nerve roots in upper and lower extremities including bilateral median, ulnar, radial, peroneal, femoral, and sciatic nerve were noted. Lumbosacral and brachial plexus roots along with sympathetic ganglia were also affected. Despite infiltration of cervical sympathetic chain, there was not evidence of symptomatic autonomic dysfunction in our patient during the course of his disease. Assessment was further complicated by associated medical complications. There was absence of lymphomatous infiltration in the brain, spinal cord, or meninges. Infiltrative lymphoma cells affected the dorsal root ganglia and spinal nerves in a patchy, irregular fashion (Figures 1A,B).

## Discussion

Neurolymphomatosis is a rare and elusive neurological complication of systemic lymphoma and leukemia. The underlying malignancy is NHL in approximately 90% of patients, with leukemia and T-cell neoplasias accounting for the rest of cases (7). Diagnosis remains challenging due to the broad spectrum of clinical presentations (2, 8–12), associated comorbidities including chemotherapy-induced side effects (13, 14), and lack of conclusive, specific findings on diagnostic testing. Four established clinical presentations of the disease and recognized, including painful infiltration of nerves or roots, cranial neuropathy with or without pain, painless involvement of peripheral nerves, and painful or painless involvement of a single peripheral nerve (2). Clinical findings suggestive of NL, as opposed to remote effects or inflammatory processes, include severe pain, asymmetric distribution, and rapid evolution (7). 18-FDG PET/CT is a non-invasive and highly sensitive diagnostic method facilitating identification of NL, as positive findings are typically highly suggestive of the diagnosis (7, 15, 16). Nevertheless, the diagnosis of NL is often difficult and depends on the histopathological identification of infiltrating malignant lymphocytes in the affected nerves typically with nerve biopsy. ^18^F-FDG uptake is highly sensitive for tumors but is not specific and abnormal uptake may also be observed in any process where the rate of glycolysis is increased. Due to the inexorable and fatal course observed in our patient, an 18-FDG PET/CT was not performed as other interventions were prioritized. Nerve biopsy should be considered in cases where diagnosis remains elusive. However, peripheral nerve involvement is irregular, and nerve biopsy may not reveal lymphoma infiltration despite neurophysiologic evidence of nerve affection (17, 18). This is in line with our patient’s presentation, as two, bilateral, sequential, sural nerve biopsies failed to demonstrate tumor infiltration. In our patient, the diagnosis of NL was made at post-mortem, which is not uncommon (2, 7). NL involves the roots within, as well as beyond, the borders of the subarachnoid space. Thus, systemic chemotherapy is critical to address the multiple sites of involvement. Unfortunately, there is no optimal therapy for NL. Several chemotherapy agents have been used achieving clinical improvement in approximately 50–70% of treated patients (7). Nevertheless, neurological symptoms may not improve despite appropriate response of the systemic lymphoma or immune-modulating therapies. CSF evaluation in our patient demonstrated only mildly elevated of the protein content without hypoglycorrhachia or evidence of lymphoma cells. The cause of selective involvement of peripheral nerve structures without meningeal or CNS involvement in our patient remains unclear. To the best of our knowledge, this is only the third case of NL affecting only the PNS. The rarity of the disease precludes additional assessments, but the possibility of a selective, immunologically mediated reaction at the blood-nerve barrier remains possible. Further studies are necessary to explore this observation in a patient with NL and lymphoma.

## Conflict of Interest Statement

The Associate Editor Gregory Gruener declares that, despite being affiliated to the same institution as authors José Biller and Sarkis Morales-Vidal, the review process was handled objectively and no conflict of interest exists.
